# Involvement of the heart in Kawasaki Disease: not always coronaritis

**DOI:** 10.1186/1546-0096-9-S1-P214

**Published:** 2011-09-14

**Authors:** MC Pellegrin, A Taddio, N Giurici, L Lepore, A Ventura

**Affiliations:** 1 IRCCS Burlo Garofolo, Department of Pediatrcs, University of Triest, Italy

## Background

Coronaritis, miocarditis, pericarditis, mitral insufficiency and pancarditis have been reported in Kawasaki Disease (KD). The aim was to evaluate all the cardiac involvement in children affectd by KD followed at out center during the past 20 years.

## Materials and Methods

The medical records of all patients diagnosed with KD at our institute between 1988 and 2010 were reviewed.

## Results

45 cases of KD were identified (64% males, mean age 32 months). Heart involvement was present in 24% (3 coronary, one of which giant aneurysm (2%), 2 pancarditis, 2 pericarditis, and 8 valvulitis). Patients with heart involvement had higher mean age, higher mean C-Reactive Protein. This subset of patients was less prone to respond to the first dose of intravenous immunoglobulins. The frequency of valvulopathy was 18%. Atrio-ventricular valves (especially mitral) presented a mild-to moderate asymptomatic insufficiency identifiable only at the echocardiography. The valvulopathy was transient, disappearing without sequelae between the second and fourth week.

## Conclusions

According to literature the rate of coronary aneurisms was 2%, while the incidence of valvular involvement appears elevated (18%). The valvular involvement is identified at the echocardiography during the acute or subacute phase, isolated or associated to miocarditis, pericarditis or coronaritis. The mitral valve appears primarily involved. Cardiac sequelae were not reported. Indeed, the valvulopathy disappeared within four weeks. These data further enhance the importance of a prompt diagnosis of KD and the need of an appropriate therapy to reduce the risk of a coronary involvement and bring to complete regression of the heart involvement.

